# CNVfilteR: an R/Bioconductor package to identify false positives produced by germline NGS CNV detection tools

**DOI:** 10.1093/bioinformatics/btab356

**Published:** 2021-05-13

**Authors:** José Marcos Moreno-Cabrera, Jesús del Valle, Elisabeth Castellanos, Lidia Feliubadaló, Marta Pineda, Eduard Serra, Gabriel Capellá, Conxi Lázaro, Bernat Gel

**Affiliations:** Hereditary Cancer Group, Program for Predictive and Personalized Medicine of Cancer, Germans Trias i Pujol Research Institute (PMPPC-IGTP), Campus Can Ruti, Badalona, 08916 Barcelona, Spain; Hereditary Cancer Program, Joint Program on Hereditary Cancer, Catalan Institute of Oncology, Institut d'Investigació Biomèdica de Bellvitge-IDIBELL, L'Hospitalet de Llobregat, 08908 Barcelona, Spain; Instituto de Salud Carlos III, Centro de Investigación Biomédica en Red Cáncer (CIBERONC), 28029 Madrid, Spain; Hereditary Cancer Program, Joint Program on Hereditary Cancer, Catalan Institute of Oncology, Institut d'Investigació Biomèdica de Bellvitge-IDIBELL, L'Hospitalet de Llobregat, 08908 Barcelona, Spain; Instituto de Salud Carlos III, Centro de Investigación Biomédica en Red Cáncer (CIBERONC), 28029 Madrid, Spain; Hereditary Cancer Group, Program for Predictive and Personalized Medicine of Cancer, Germans Trias i Pujol Research Institute (PMPPC-IGTP), Campus Can Ruti, Badalona, 08916 Barcelona, Spain; Clinical Genomics Unit, Clinical Genetics Service, Northern Metropolitan Clinical Laboratory, Germans Trias i Pujol University Hospital (HUGTiP), Campus Can Ruti, Badalona, 08916 Barcelona, Spain; Hereditary Cancer Program, Joint Program on Hereditary Cancer, Catalan Institute of Oncology, Institut d'Investigació Biomèdica de Bellvitge-IDIBELL, L'Hospitalet de Llobregat, 08908 Barcelona, Spain; Instituto de Salud Carlos III, Centro de Investigación Biomédica en Red Cáncer (CIBERONC), 28029 Madrid, Spain; Hereditary Cancer Program, Joint Program on Hereditary Cancer, Catalan Institute of Oncology, Institut d'Investigació Biomèdica de Bellvitge-IDIBELL, L'Hospitalet de Llobregat, 08908 Barcelona, Spain; Instituto de Salud Carlos III, Centro de Investigación Biomédica en Red Cáncer (CIBERONC), 28029 Madrid, Spain; Hereditary Cancer Group, Program for Predictive and Personalized Medicine of Cancer, Germans Trias i Pujol Research Institute (PMPPC-IGTP), Campus Can Ruti, Badalona, 08916 Barcelona, Spain; Instituto de Salud Carlos III, Centro de Investigación Biomédica en Red Cáncer (CIBERONC), 28029 Madrid, Spain; Hereditary Cancer Program, Joint Program on Hereditary Cancer, Catalan Institute of Oncology, Institut d'Investigació Biomèdica de Bellvitge-IDIBELL, L'Hospitalet de Llobregat, 08908 Barcelona, Spain; Instituto de Salud Carlos III, Centro de Investigación Biomédica en Red Cáncer (CIBERONC), 28029 Madrid, Spain; Hereditary Cancer Program, Joint Program on Hereditary Cancer, Catalan Institute of Oncology, Institut d'Investigació Biomèdica de Bellvitge-IDIBELL, L'Hospitalet de Llobregat, 08908 Barcelona, Spain; Instituto de Salud Carlos III, Centro de Investigación Biomédica en Red Cáncer (CIBERONC), 28029 Madrid, Spain; Hereditary Cancer Group, Program for Predictive and Personalized Medicine of Cancer, Germans Trias i Pujol Research Institute (PMPPC-IGTP), Campus Can Ruti, Badalona, 08916 Barcelona, Spain

## Abstract

**Summary:**

Germline copy-number variants (CNVs) are relevant mutations for multiple genetics fields, such as the study of hereditary diseases. However, available benchmarks show that all next-generation sequencing (NGS) CNV calling tools produce false positives. We developed CNVfilteR, an R package that uses the single-nucleotide variant calls usually obtained in germline NGS pipelines to identify those false positives. The package can detect both false deletions and false duplications. We evaluated CNVfilteR performance on callsets generated by 13 CNV calling tools on three whole-genome sequencing and 541 panel samples, showing a decrease of up to 44.8% in false positives and consistent F1-score increase. Using CNVfilteR to detect false-positive calls can improve the overall performance of existing CNV calling pipelines.

**Availability and implementation:**

CNVfilteR is released under Artistic-2.0 License. Source code and documentation are freely available at Bioconductor (http://www.bioconductor.org/packages/CNVfilteR).

**Supplementary information:**

Supplementary data are available at *Bioinformatics* online.

## 1 Introduction

Copy-number variants (CNVs) are a type of structural variant which has been a matter of interest in multiple genetic fields. In the research and diagnosis of hereditary diseases, where CNVs are relevant contributors (Zhang *et al.*, 2019), the analysis of germline CNVs plays a key role. Recent improvements in next-generation sequencing (NGS) have resulted in the release of several tools for germline CNV detection on whole-genome sequencing (WGS), whole-exome sequencing and panel data (Mason-Suares *et al.*, 2016; Roca *et al.*, 2019; Zhao *et al.*, 2013). Nevertheless, CNV detection in NGS is challenging due to aspects relative to the technology, such as short-read lengths or GC-content bias (Teo *et al.*, 2012).

Available benchmarks show that all germline CNV calling tools produce false positives (Kim *et al.*, 2017; Moreno-Cabrera *et al.*, 2020; Zhang *et al.*, 2019), frequently reaching high false discovery rates (FDRs). These false-positive calls impact downstream analysis. In a clinical setting, where the use of an orthogonal method is necessary to validate a CNV, false-positive calls lead laboratories to make an important effort to validate them. A tool able to identify these false-positive calls could help in this regard.

Most NGS CNV callers are based on one or more of these strategies: read-pair, split-read, read-depth and assembly based (Pirooznia *et al.*, 2015). However, information from single-nucleotide variants (SNVs), usually available in NGS pipelines, is rarely used in CNV detection strategies although SNV allele frequency can provide evidence to confirm or discard CNV calls.

Here, we present CNVfilteR, an R/Bioconductor package that uses SNVs to identify false positives in the output of CNV calling tools.

## 2 False-positive identification strategy

**Fig. 1.**
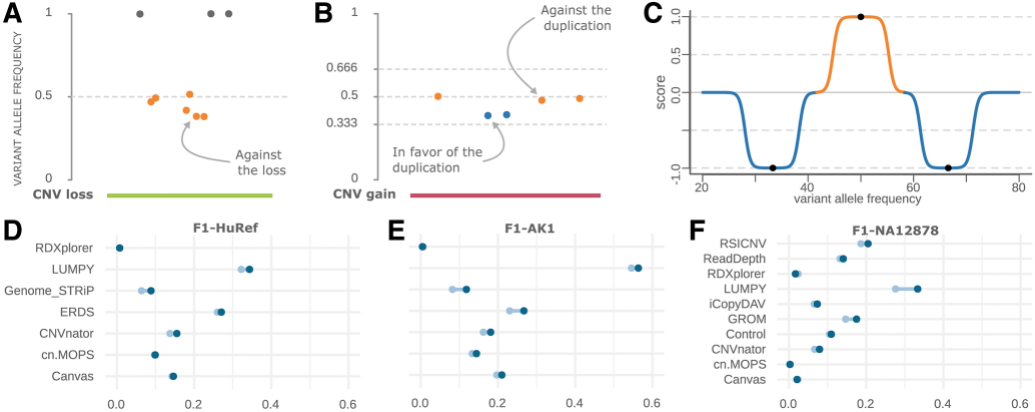
(**A**) CNV deletion example, adapted from CNVfilteR output. (**B**) CNV duplication example, adapted from CNVfilteR output. (**C**) Scoring model for CNV duplications, plotted by CNVfilteR. (**D–F**) F1-score differences before (light blue) and after (dark blue) removing the false-positive CNVs identified by CNVfilteR in the HuRef, AK1 and NA12878 WGS samples

CNVfilteR uses two different strategies to identify false-positives CNV calls in diploid genomes. Heterozygous deletions are loss-of-heterozygosity regions and cannot overlap with heterozygous SNVs, since only one allele remains. If a heterozygous SNV is detected within a deleted region, either the SNV or the deletion is a false positive (Fig. 1a). To account for errors in SNV calling, a CNV deletion is identified as false positive if at least a percentage of the SNVs overlapping that CNV is heterozygous, 30% by default. On the other hand, CNV duplications are evaluated using a fuzzy-logic-inspired model which scores all heterozygous SNVs overlapping the CNV. If the duplication was a true-positive, the expected allele frequency of heterozygous SNVs would be either 33% or 66%, while it would be 50% if the duplication was a false positive (Fig. 1b). Therefore, each SNV is scored with a value between −1 and 1 depending on how close the allele frequency is to the nearest expected allele frequency (Fig. 1c). If the sum of the scores of all the SNVs in the CNV is greater than the duplication threshold value, the CNV duplication is identified as false positive. Further details of the scoring model can be found in Supplementary File S1.

## 3 Features

### 3.1 Input formats

VCF format is the most common output of SNV callers and its interpretation is challenging due to the flexibility provided by the format specification. CNVfilteR provides a function to interpret automatically VCFs produced by VarScan2, Strelka/Strelka2, freeBayes, HaplotypeCaller (GATK) and UnifiedGenotyper (GATK). Output from other tools can also be loaded if adequate parameters are provided.

### 3.2 Visual output

Results can be plotted and customized through plotting functions based on karyoploteR (Gel and Serra, 2017) and CopyNumberPlots (https://github.com/bernatgel/CopyNumberPlots) packages (Supplementary Fig. S1).

## 4 Performance evaluation

CNVfilteR was evaluated on 3 WGS samples and 541 gene-panel samples. The default parameters were chosen based on their performance in a WGS sample (HuRef sample) and a gene-panel dataset (HiSeq-panel) (Supplementary File S1).

### 4.1 Evaluation on WGS data

We evaluated CNVfilteR performance on three reference WGS samples: the HuRef/Venter genome (Zhou *et al.*, 2018), the AK1 genome (Seo *et al.*, 2016) and the NA12878 genome. The HuRef and AK1 samples were evaluated using a published reference CNV callset and the results of six CNV calling tools (Canvas, cn. MOPS, CNVnator, ERDS, Genome_STRiP, RDXplorer) (Trost *et al.*, 2018). For these two samples, we also ran an additional CNV calling tool, LUMPY (Layer *et al.*, 2014). On the other hand, we evaluated the NA12878 sample with a reference callset and the output of ten CNV calling tools (Canvas, cn. MOPS, CNVnator, RDXplorer, iCopyDAV, GROM-RD, Rsicnv, Control-FREEC, ReadDepth) from a previous work (MacDonald *et al.*, 2014; Parikh *et al.*, 2016; Zhang *et al.*, 2019). For the three WGS samples, SNV calls were obtained using Strelka2 (Kim *et al.*, 2018). Further details are available in Supplementary File S1.

CNVfilteR identified between 15.3% and 44.8% of the false positives and the FDR decreased for all tool-sample evaluations (up to 10.4%). Additionally, F1-score was improved in 19 out of the 24 tool-sample evaluations reaching up to 20.7% F1-score increase (Fig. 1d–f). Sensitivity, however, decreased slightly: tool-sample evaluations had an absolute sensitivity decrease between 0.001 and 0.035. Metrics details are available in Supplementary File S2 and Figures S2–S7. Moreover, additional evaluations were performed to show CNVfilteR performance on different CNV size ranges, on different number of SNVs overlapping each CNV, and on different parameter values (Supplementary Figs S8–S25 and Files S5–S7).

### 4.2 Evaluation on gene-panel data

We also evaluated CNVfilteR performance on two gene-panel targeted datasets: one containing 411 samples from different Illumina HiSeq runs (HiSeq-panel dataset) and another with 130 samples from different Illumina MiSeq runs (MiSeq-panel dataset). All samples were captured with a 135-gene panel (Castellanos *et al.*, 2017). To evaluate CNVfilteR, previous MLPA results for a subset of genes were used as gold-standard, CNVs were called using DECoN (Fowler *et al.*, 2016), and SNVs were called using VarScan2 (Koboldt *et al.*, 2012) (Supplementary Files S1, S3 and S4).

In the HiSeq-panel and MiSeq-panel datasets, CNVfilteR identified 15% of the false-positive calls (3 out of 20 false positives) and 12.5% of the false-positive calls (2 out of 16), respectively.

On both datasets, no true CNV was misidentified as false positive (Supplementary File S1), so sensitivity did not change.

### 4.3 Runtime

Runtime was evaluated on a subset of 79 gene-panel samples and the HuRef WGS sample. The median runtime per sample was 0.85 s for the gene-panel samples and 3.53 min for the HuRef sample (Supplementary File S1).

## 5 Conclusion

We developed CNVfilteR, an R/Bioconductor package to identify false-positive calls generated by CNV calling tools from germline NGS data using SNVs’ allele frequency. CNVfilteR identified false-positive calls in all tested tools and datasets, from gene-panel to WGS, and F1-score was improved in most tool-sample combinations. CNVfilteR can be plugged in most existing CNV calling pipelines to improve calling performance at virtually no cost.

## Supplementary Material

btab356_Supplementary_Data
