# Supplementary material for: CNVfilteR: an R/Bioconductor package to identify false positives produced by germline NGS CNV detection tools
Source: Bioinformatics. 2021 May 13;37(22):4227–9. doi: 10.1093/bioinformatics/btab356 (PMC9502136; doi:10.1093/bioinformatics/btab356)
Supplement: btab356_Supplementary_Data [file btab356_supplementary_data.zip › SuppFigures.docx]

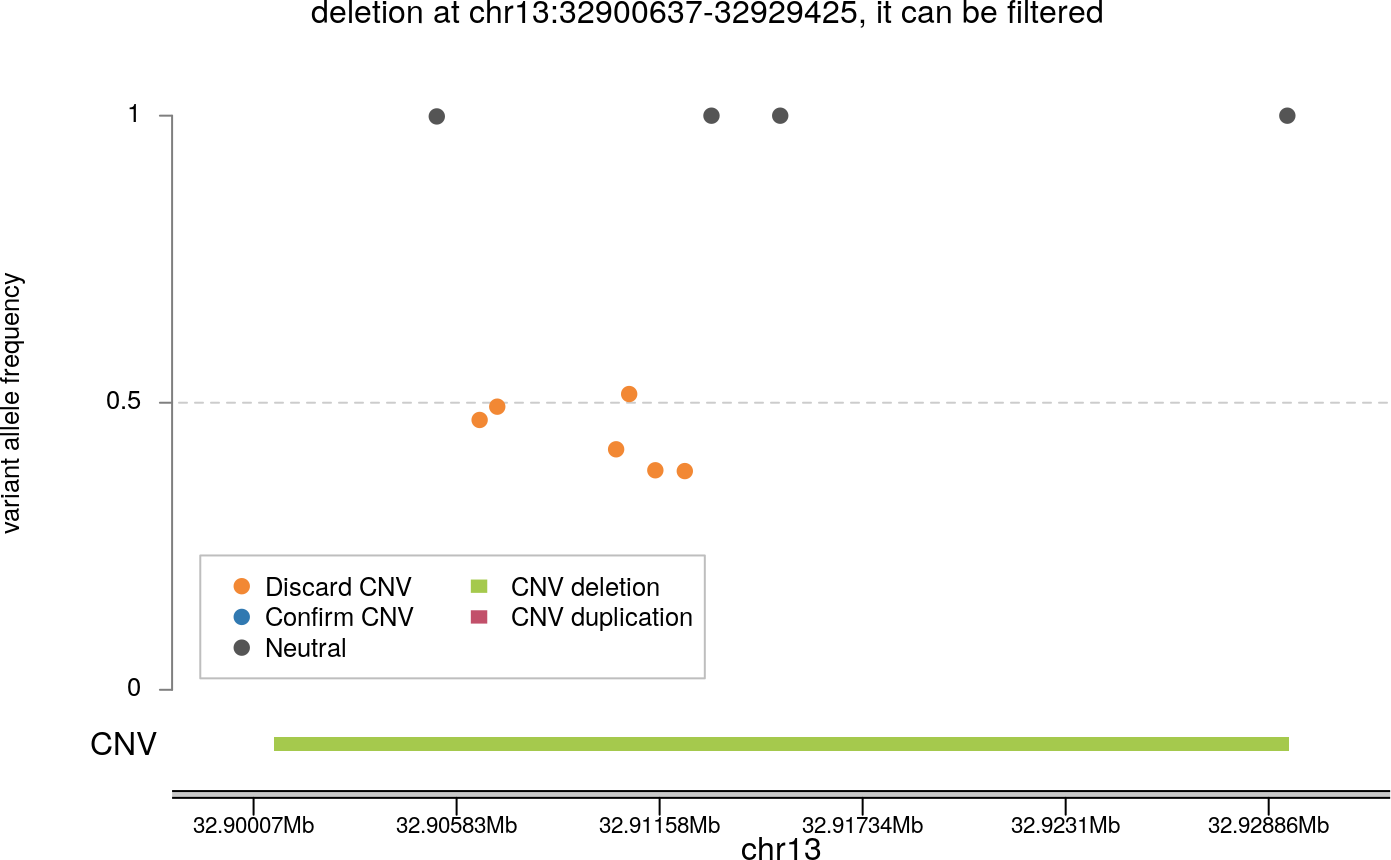


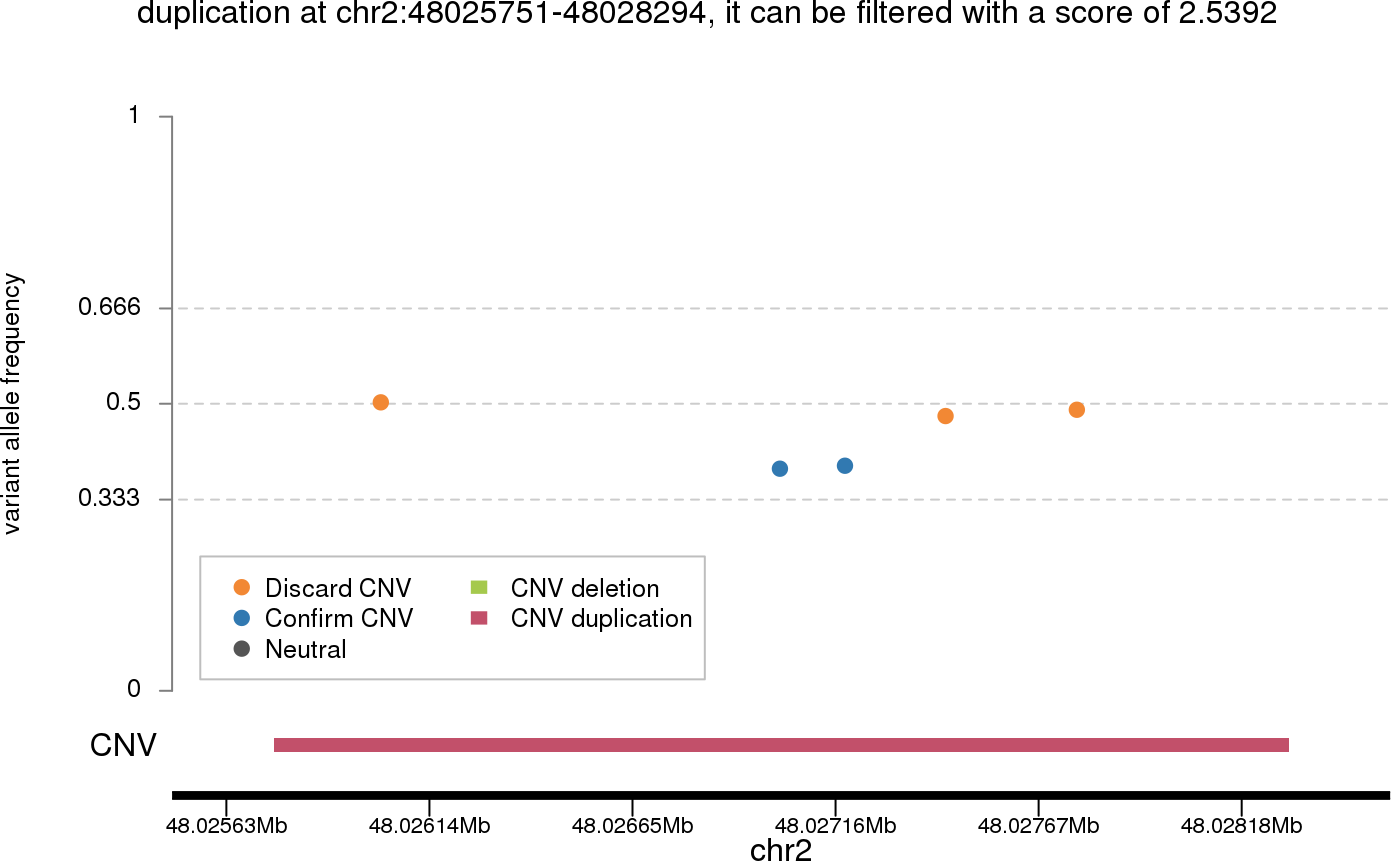


**Supplementary Figure 1:** CNVfilteR output examples for a CNV deletion and a CNV duplication.

In the first example, six heterozygous SNVs overlapped the CNV deletion, providing evidence to identify it as a false-positive CNV (so it could be filtered out). In the second example, five heterozygous SNVs overlapped the CNV duplication: three out of them with an allele frequency very close to 0.5 and two out of them near – but not close – to 0.33. In this second example, adding up the total scores provided by the CNVfilteR scoring model, the final score was higher than the duplication threshold score (0.5), so the CNV was identified as a false positive and could be discarded.

**
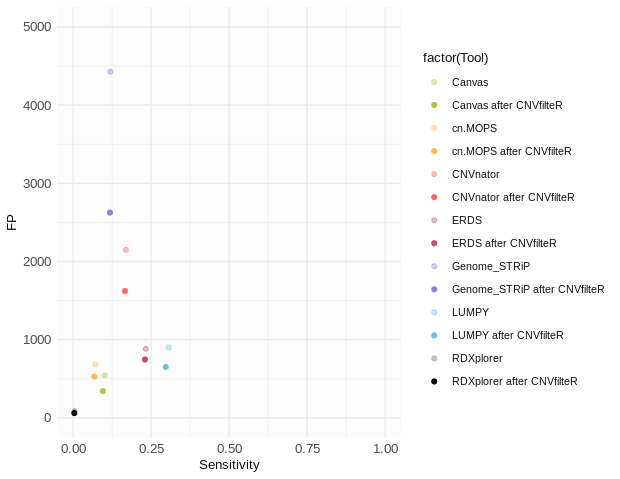
**

**Supplementary Figure 2:** Sensitivity and false positives (FP) for the HuRef sample.

**
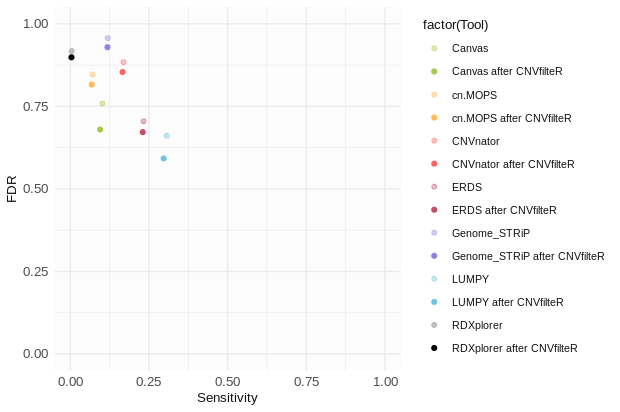
**

**Supplementary Figure 3:** Sensitivity and false discovery rate (FDR) for the HuRef sample.


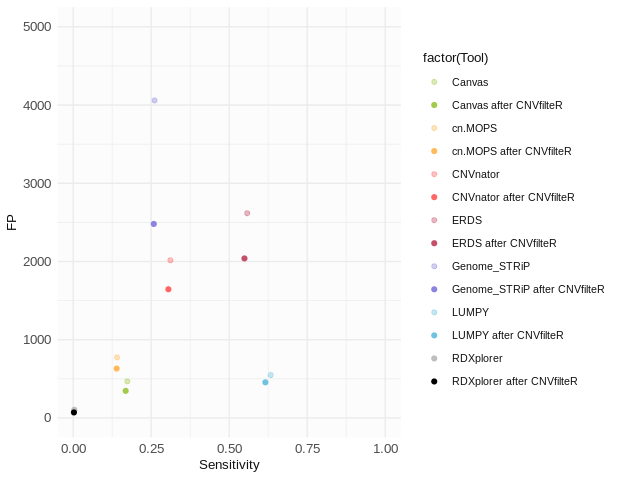


**Supplementary Figure 4:** Sensitivity and false positives (FP) for the AK1 sample.

**
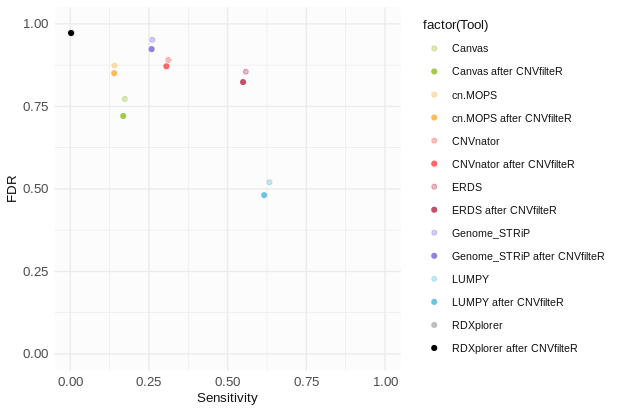
**

**Supplementary Figure 5:** Sensitivity and false discovery rate (FDR) for the AK1 sample.

**
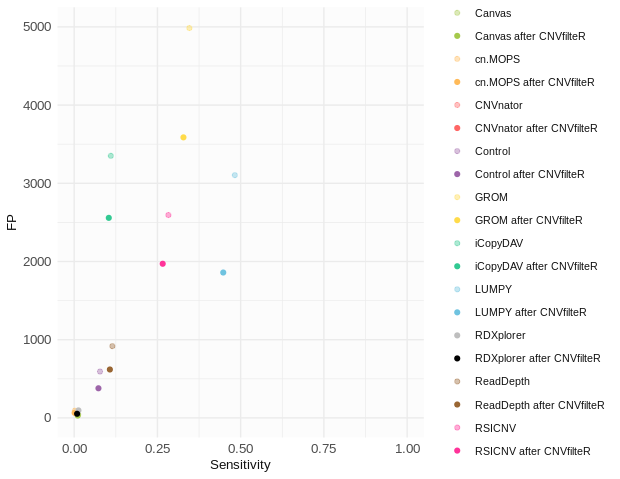
**

**Supplementary Figure 6:** Sensitivity and false positives (FP) for the NA12878 sample.


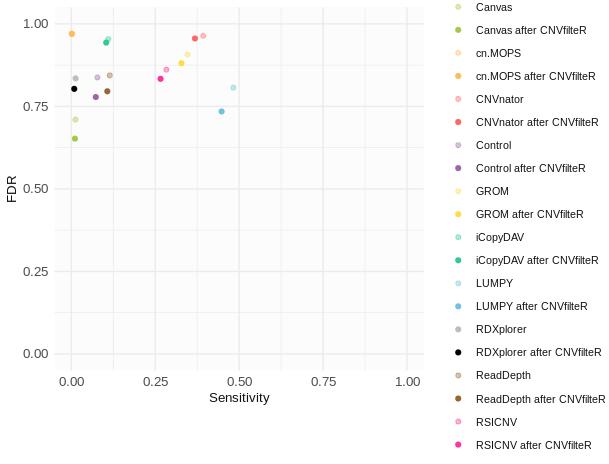


**Supplementary Figure 7:** Sensitivity and false discovery rate (FDR) for the NA12878 sample.


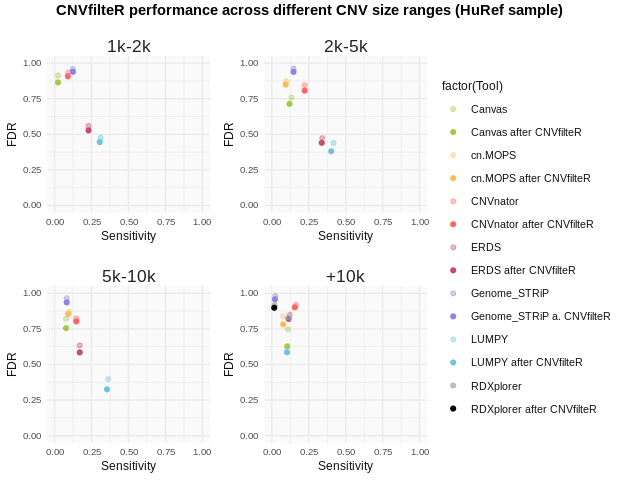


**Supplementary Figure 8:** Sensitivity and false discovery rate (FDR) by event size for the HuRef sample. 1k-2k: 1000 to 1999 bp; 2k-5k: 2000 to 4999 bp; 5k-10k: 5000 to 9999 bp; +10k: ≥ 10000 bp. RDXplorer only called CNVs in the +10k range and cn.MOPS did no call any CNV in the 1k-2k range.


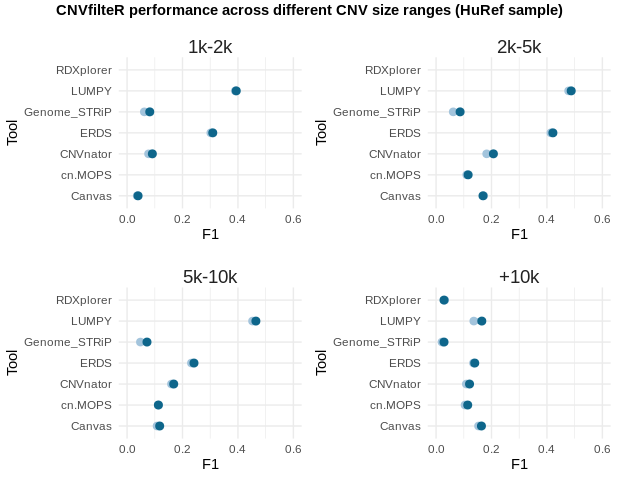


**Supplementary Figure 9:** F1-score by event size for the HuRef sample. 1k-2k: 1000 to 1999 bp; 2k-5k: 2000 to 4999 bp; 5k-10k: 5000 to 9999 bp; +10k: ≥ 10000 bp. RDXplorer only called CNVs in the +10k range and cn.MOPS did no call any CNV in the 1k-2k range.


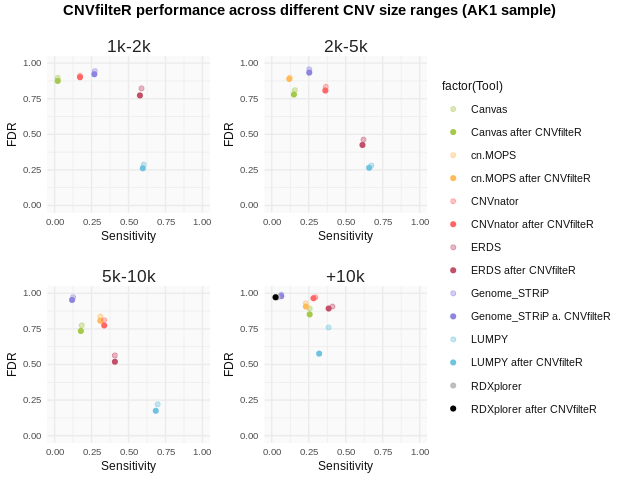


**Supplementary Figure 10:** Sensitivity and false discovery rate (FDR) by event size for the AK1 sample. 1k-2k: 1000 to 1999 bp; 2k-5k: 2000 to 4999 bp; 5k-10k: 5000 to 9999 bp; +10k: ≥ 10000 bp. RDXplorer only called CNVs in the +10k range and cn.MOPS did no call any CNV in the 1k-2k range.


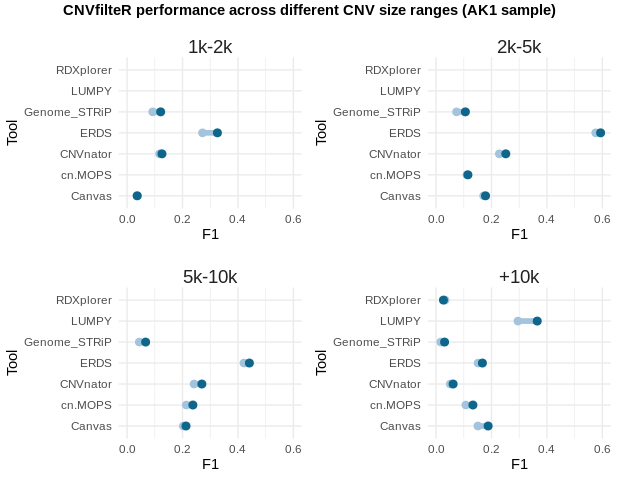


**Supplementary Figure 11:** F1-score by event size for the AK1 sample. 1k-2k: 1000 to 1999 bp; 2k-5k: 2000 to 4999 bp; 5k-10k: 5000 to 9999 bp; +10k: ≥ 10000 bp. RDXplorer only called CNVs in the +10k range and cn.MOPS did no call any CNV in the 1k-2k range.


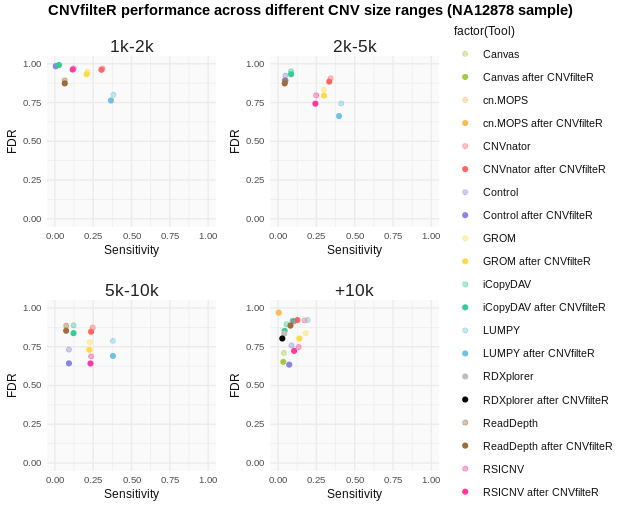


**Supplementary Figure 12:** Sensitivity and false discovery rate (FDR) by event size for the NA12878 sample. 1k-2k: 1000 to 1999 bp; 2k-5k: 2000 to 4999 bp; 5k-10k: 5000 to 9999 bp; +10k: ≥ 10000 bp. RDXplorer, cn.MOPS and Canvas only called CNVs in the +10k range.


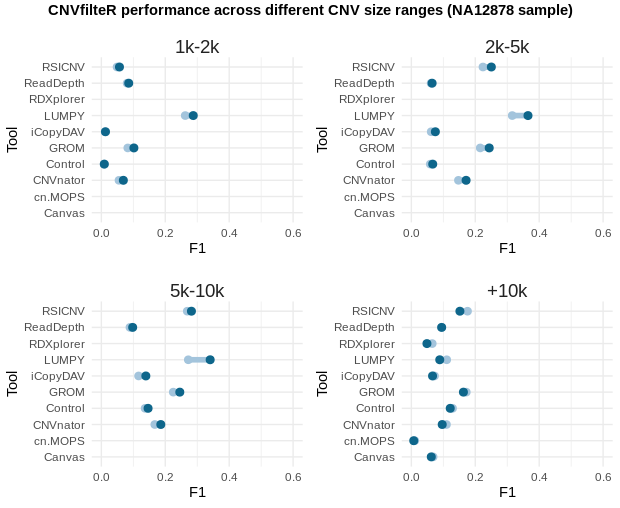


**Supplementary Figure 13:** F1-score by event size for the NA12878 sample. 1k-2k: 1000 to 1999 bp; 2k-5k: 2000 to 4999 bp; 5k-10k: 5000 to 9999 bp; +10k: ≥ 10000 bp. RDXplorer, cn.MOPS and Canvas only called CNVs in the +10k range.


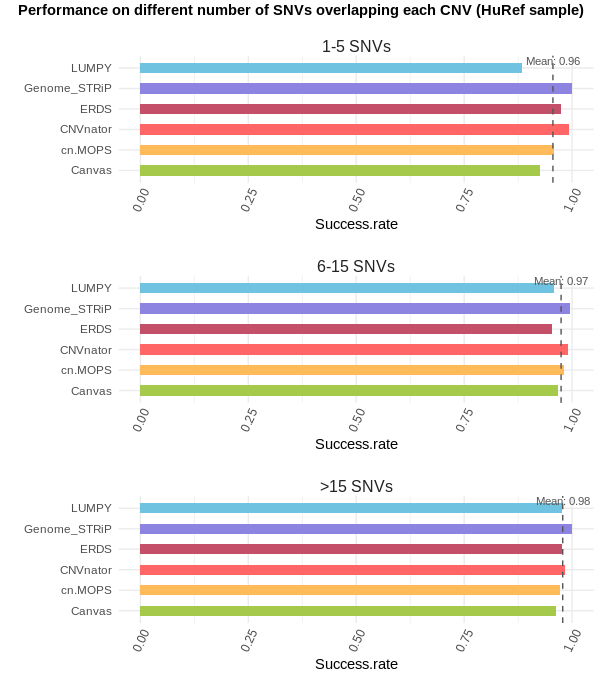


**Supplementary Figure 14:** CNVfilteR performance when the number of SNVs spanning a CNV varies for the HuRef sample. The *Success.rate* variable measures the CNVfilteR success when identifying a CNV call as a false positive: success if the CNV call did not overlap any CNV in the reference dataset, failure otherwise. *Success.rate*: number of success / (number of success + number of failures). Tools having less than 10 values in any SNVs range (RDXexplorer) were omitted.


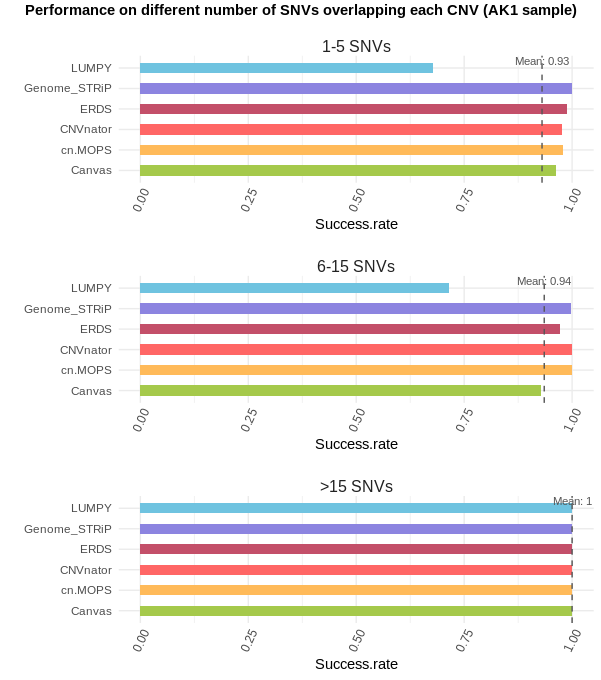


**Supplementary Figure 15:** CNVfilteR performance when the number of SNVs spanning a CNV varies for the AK1 sample. The *Success.rate* variable measures the CNVfilteR success when identifying a CNV call as a false positive: success if the CNV call did not overlap any CNV in the reference dataset, failure otherwise. *Success.rate*: number of success / (number of success + number of failures). Tools having less than 10 values in any SNVs range (RDXexplorer) were omitted.


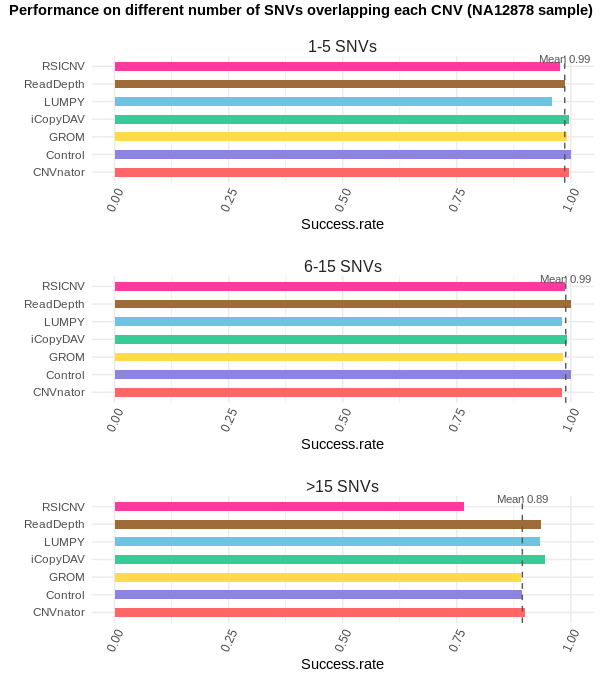


**Supplementary Figure 16:** CNVfilteR performance when the number of SNVs spanning a CNV varies for the NA12878 sample. The *Success.rate* variable measures the CNVfilteR success when identifying a CNV call as a false positive: success if the CNV call did not overlap any CNV in the reference dataset, failure otherwise. *Success.rate*: number of success / (number of success + number of failures). Tools having less than 10 values in any SNVs range (RDXexplorer, cn.MOPS, Canvas) were omitted. Although AK1 and HuRef showed an increasing success rate when the number of SNVs overlapping each CNV grew, performance decreased for the NA12878 in the >15 SNVs group.


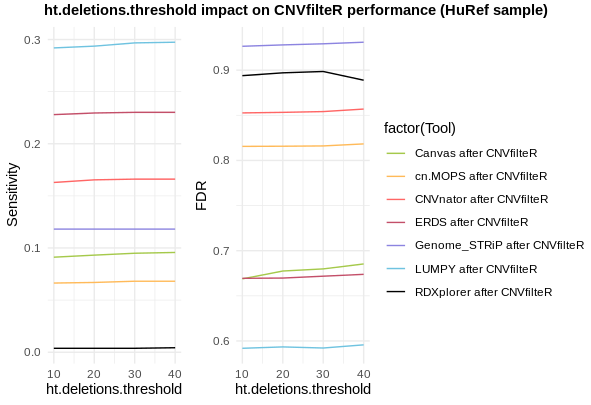


**Supplementary Figure 17:** CNVfilteR sensitivity and false discovery rate (FDR) when varying the ht.deletions.threshold parameter value for the HuRef sample. All other parameters were executed with their default values.


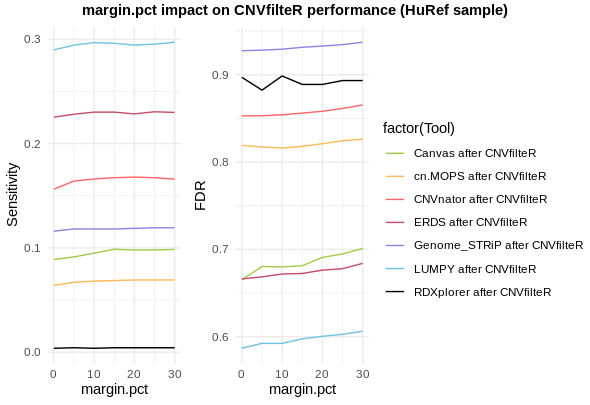


**Supplementary Figure 18:** CNVfilteR sensitivity and false discovery rate (FDR) when varying the margin.pct parameter value for the HuRef sample. All other parameters were executed with their default values.


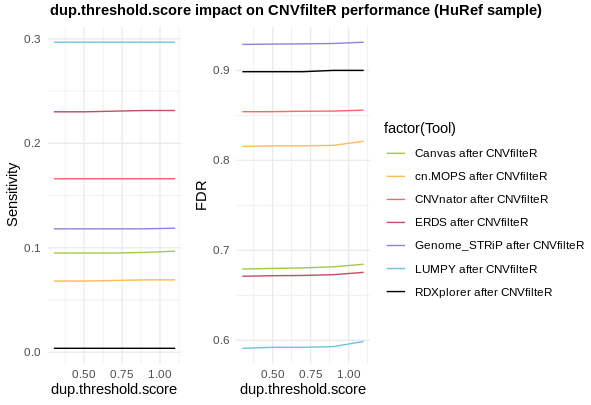


**Supplementary Figure 19:** CNVfilteR sensitivity and false discovery rate (FDR) when varying the dup.threshold.score parameter value for the HuRef sample. All other parameters were executed with their default values.


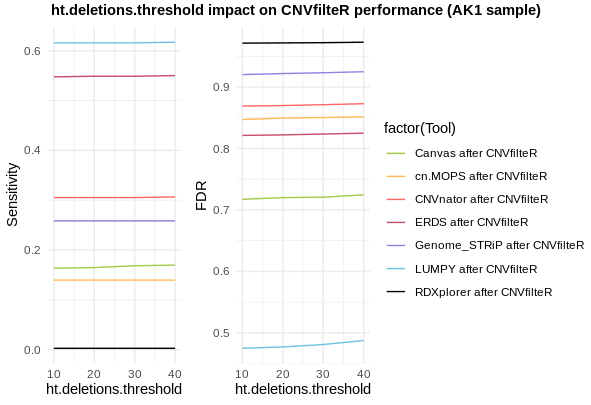


**Supplementary Figure 20:** CNVfilteR sensitivity and false discovery rate (FDR) when varying the ht.deletions.threshold parameter value for the AK1 sample. All other parameters were executed with their default values.


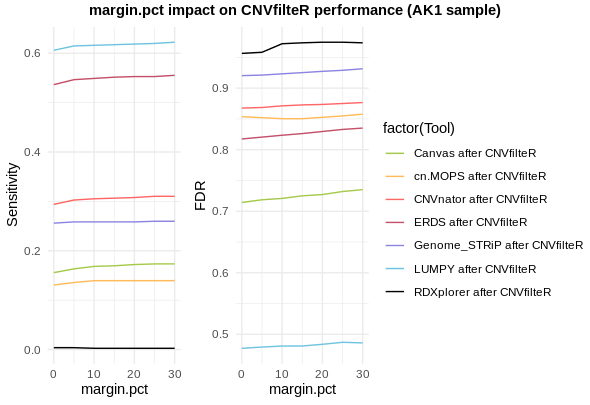


**Supplementary Figure 21:** CNVfilteR sensitivity and false discovery rate (FDR) when varying the margin.pct parameter value for the AK1 sample. All other parameters were executed with their default values.


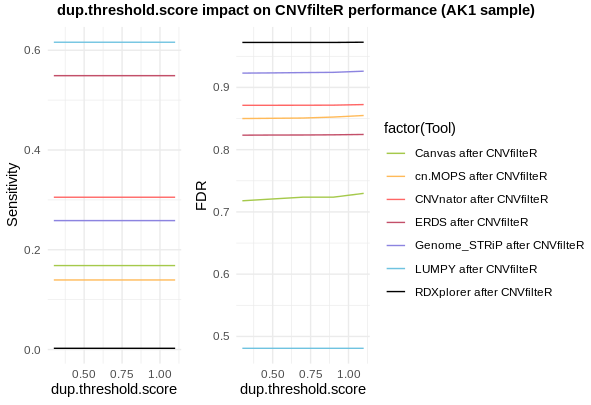


**Supplementary Figure 22:** CNVfilteR sensitivity and false discovery rate (FDR) when varying the dup.threshold.score parameter value for the AK1 sample. All other parameters were executed with their default values.


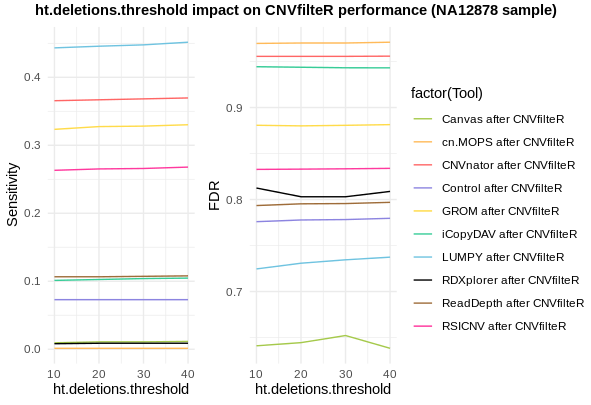


**Supplementary Figure 23:** CNVfilteR sensitivity and false discovery rate (FDR) when varying the ht.deletions.threshold parameter value for the NA12878 sample. All other parameters were executed with their default values.


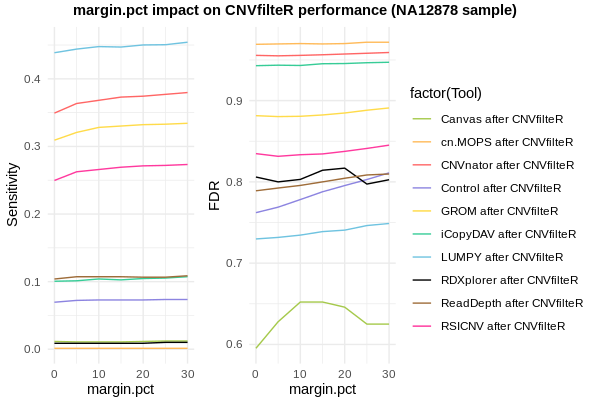


**Supplementary Figure 24:** CNVfilteR sensitivity and false discovery rate (FDR) when varying the margin.pct parameter value for the NA12878 sample. All other parameters were executed with their default values.


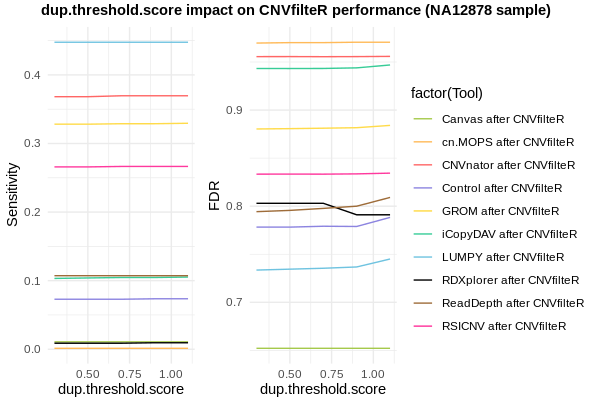


**Supplementary Figure 25:** CNVfilteR sensitivity and false discovery rate (FDR) when varying the dup.threshold.score parameter value for the NA12878 sample. All other parameters were executed with their default values.
