# Supplementary material for: CNVfilteR: an R/Bioconductor package to identify false positives produced by germline NGS CNV detection tools
Source: Bioinformatics. 2021 May 13;37(22):4227–9. doi: 10.1093/bioinformatics/btab356 (PMC9502136; doi:10.1093/bioinformatics/btab356)
Supplement: btab356_Supplementary_Data [file btab356_supplementary_data.zip › SuppFile1.docx]

**Scoring model for CNV duplications**

CNVfilteR identifies a certain CNV duplication as a false positive using the allele frequency of the heterozygous SNVs in that CNV. Each SNV is scored using a scoring model, and if the sum of the scores of all the SNVs in the CNV is greater than the duplication threshold score (defaults to 0.5), the CNV is identified as false positive. The scoring model is based on fuzzy logic, where elements can have any value between 1 (True) and 0 (False). A common way of applying fuzzy logic is using the sigmoid function. CNVfilteR uses the sigmoid function implemented in the pracma package, which is defined as y = 1 / (1 + e ^ (−c1(x − c2))). The scoring model is built on 6 sigmoids defined on 6 different intervals. The c1 parameter is 2 by default, and the c2 parameter is defined for the 6 sigmoids:

- First sigmoid: interval [20, 33.3], c2=28
- Second sigmoid: interval [33.3, 41.65], c2=38.3
- Third sigmoid: interval [41.65, 50], c2=44.7
- Fourth sigmoid: interval [50, 58.3], c2=55.3
- Fifth sigmoid: interval [58.3, 66.6], c2=61.3
- Sixth sigmoid: interval [66.6, 80], c2=71.3


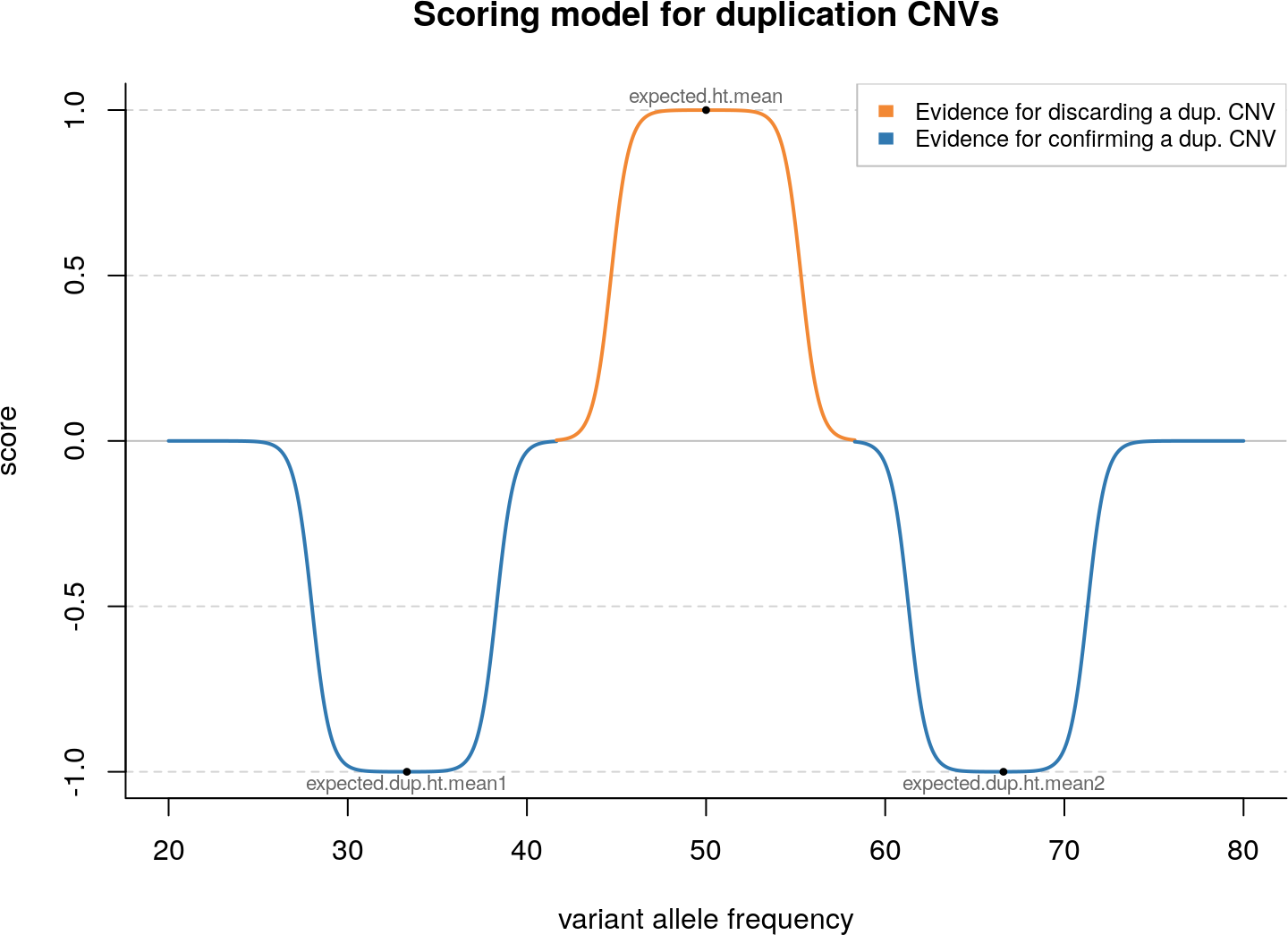


All parameter values are customizable. Code examples of how to plot and modify the scoring model are available at [Bioconductor site](https://bioconductor.org/packages/release/bioc/vignettes/CNVfilteR/inst/doc/CNVfilteR.html#scoring-model-for-duplication-cnvs).

**Evaluation on HuRef, AK1 and NA12878 samples**

Data, tools and evaluation metrics

CNVfilteR was evaluated on the HuRef, AK1 and NA12878 genomes. Reference callsets and CNV calls from different tools were obtained from different sources (see table below).

To obtain the SNV calls for each sample, they were downloaded and aligned to the hs37d5 human genome assembly using BWA mem v0.7.13. SAMtools v0.1.8 was used to sort and index BAM files and duplicates were marked using Picard v2.18.4. Point mutations were called with Strelka v2.9.3. To enrich the CNV tools results for the HuRef and AK1 genomes, LUMPY v0.2.13 (via smoove v0.2.3) was also executed to call CNVs. Details are summarized in the following table.

|  | **Reference call set** | **CNV tools results** | **SRA accession number** |
| --- | --- | --- | --- |
| **HuRef** | Obtained from Trost et al. 2018 (file S1). Contains deletions and duplications ≥1 kb. | Obtained from Trost et al. 2018 (file S5). LUMPY results were obtained from our own pipeline as explained above. Only calls ≥ 500 bp were retained. | SRR7097859 |
| **AK1** | Obtained from Trost et al. 2018, (file S4), which used Seo et al. 2016 as source. Contains only deletions ≥1 kb. | Same as the HuRef sample. Only deletions ≥ 500 bp were retained. | SRR3602759 |
| **NA12878** | Provided by Zhang et al. 2019 authors, which used Parikh et al 2016 and MacDonald et al. 2014 as sources. Contains deletions and duplications > 1 kb. A similar version without the CNV type is also available at Zhang et al. 2019 publication. | Provided by Zhang et al. 2019. Only calls ≥ 500 bp were retained. A similar version without the CNV type is also available at Zhang et al. 2019 publication. | SRR622457 |

SRA: Sequence Read Archive from NCBI

Evaluation metrics

A tool call was defined as true positive (TP) if it had a 50% reciprocal overlap with any reference call, false positive (FP) otherwise. If a certain reference call had no reciprocal overlap with any tool call, it was counted as false negative (FN). Sensitivity was defined as TP / (TP + FN), false discovery rate as FP / (FP + TP) and F1-score as 2TP / (2TP + FP + FN).

**Evaluation on HiSeq-panel and MiSeq-panel datasets**

Datasets and tools

CNVfilteR was evaluated on 541 gene-panel samples (411 HiSeq and 130 MiSeq samples, see table below), which are a superset of the samples used in a previous work (Moreno-Cabrera *et al*. 2020). Both HiSeq and MiSeq datasets contained data from a hybridization-based target capture NGS panel, called I2HCP, designed for hereditary cancer diagnostics (Castellanos *et al*., 2017). Both datasets were generated in real diagnostics settings and contained single and multi-exon CNVs, all of them validated by MLPA. Negative MLPA data, meaning no detection of any CNV, was also available for a subset of genes. Detailed information on MLPA-detected CNVs for each dataset can be found in Supplementary File 3. Samples were generated at the ICO-IGTP Joint Program for Hereditary Cancer. All MiSeq samples and a subset of HiSeq samples are available at the EGA under the accession number EGAS00001004316. All samples were aligned to the GRCh37 human genome assembly using BWA mem v0.7.12. SAMtools v0.1.19 was used to sort and index BAM files. No additional processing or filtering was applied to the BAM files. Varscan v2.4.1 was used to call point mutations and DECoN v1.0.1 was chosen for calling CNVs.

|  | **Samples** | **Validated genes with CNV** | **Single-exon CNVs** | **Multi-exon CNVs** | **Deletion CNVs** | **Duplication CNVs** | **Validated genes with no CNV** |
| --- | --- | --- | --- | --- | --- | --- | --- |
| **MiSeq dataset** | 130 | 64 | 19 | 45 | 56 | 8 | 167 |
| **HiSeq dataset** | 411 | 62 | 19 | 43 | 52 | 10 | 1076 |

Regions of interest

We generated a target bed file containing all coding exons from all protein-coding transcripts of genes in the I2HCP panel v2.1 (Supplementary File 4). This data was retrieved from Ensembl Biomart version 67 may2012.archive.ensembl.org). All genes tested by MLPA and used in the benchmark were common to all I2HCP versions (v2.0-2.2).

Evaluation metrics

Performance metrics were performed per gene given that most MLPA kits cover a whole gene and so the true CNVs would be detected by MLPA when confirming any CNV call in any region of interest (ROI) of the affected gene. Therefore, a CNV tool call was defined as TP if one of its ROIs was a TP; FN if MLPA detected a CNV in at least one of its ROIs and none of them were detected by the tool; FP if the tool called a CNV in at least one ROI and none of them were detected by MLPA; TN if neither MLPA or the tool detected a CNV in any of its ROIs.

**Runtime**

Runtime was calculated by executing CNVfilteR ten times on a dataset of 79 gene-panel samples and on the HuRef WGS sample. The runtime calculations were performed on an Intel i5‐2450M CPU (4 cores, 2.50 GHz) with 8 GB of RAM and an SSD disk. The median runtime per sample was 0.84 seconds for the gene-panel samples, and 3.60 minutes for the HuRef sample. See the table below for more details.

|  | **Gene-panel samples** | **HuRef WGS sample** |
| --- | --- | --- |
| Number of samples evaluated | 79 samples evaluated at once | 1 |
| Number of variants per sample | 1554.3 (From VarScan) | 4602440 (From Strelka) |
| Number of CNVs per sample | 0.55 (From DECoN) | 1362 (From LUMPY) |
| Total runtime (median value) | 66.57 seconds | 3.53 minutes |
| Runtime per sample (median value) | 0.84 seconds | 3.53 minutes |

**Summary of CNVfilteR parameters**

| **Parameter** | **Description** | **Value used on WGS evaluation (default values)** | **Value used on gene-panel data evaluation** |
| --- | --- | --- | --- |
| *ht.deletions.threshold* | Minimum percentage of heterozygous SNVs in a CNV deletion to filter that CNV | 30 | = |
| *min.total.depth* | SNV minimum total depth | 10 | 30 |
| *dup.threshold.score* | A CNV duplication is identified as false positive if the sum of the scores of all the heterozygous SNVs in the CNV is equal or greater than the *dup.threshold.score* limit. | 0.5 | = |
| *margin.pct* | Percentage of CNV length, from each CNV limit, where SNVs will be ignored | 10 | 0 |
| *homozygous.range* | Allele frequency interval at which SNVs are considered homozygous. | [90-100] | = |
| *heterozygous.range* | Allele frequency interval at which SNVs are considered heterozygous | [28-72] | = |
| *expected.ht.mean* | Expected heterozygous SNV allele frequency | 50 | = |
| *expected.dup.ht.mean1* | Expected heterozygous SNV allele frequency when the variant IS NOT in the same allele as the CNV duplication | 33.3 | = |
| *expected.dup.ht.mean2* | Expected heterozygous SNV allele frequency when the variant IS in the same allele as the CNV duplication | 66.6 | = |
| *sigmoid.c1* | Sigmoid c1 parameter | 2 | = |
| *sigmoid.c2.vector* | Vector containing sigmoid c2 parameters for the six sigmoid functions | (28, 38.3, 44.7, 55.3, 61.3, 71.3) | = |

Two parameter values were slightly modified for the gene-panel data evaluation. We used a *min.total.depth* value of 10 to fit better the sample coverage and the SNV caller used (VarScan), and a *margin.pct* of 0 because of the small windows (regions of interest) used in gene-panel data.

**CNVfilteR use recommendations**

CNVfilteR uses SNVs to identify false-positive CNV calls. Therefore, its performance depends on the SNV calls quality. Some considerations can be followed in order to provide reliable SNVs to CNVfilteR:

- Low complexity and repetitive regions are genome areas where SNV callers (also CNV callers) perform poorly. If possible, ignore these regions when using CNVfilteR.
- Use the *min.depth* parameter to discard SNVs with low depth coverage. The default value is 10, which may be appropriate in many WGS samples, but this value should be adapted to your experiment conditions.
- Many CNV callers produce inaccurate CNV calls. These inaccurate CNV calls are more likely to be true (to overlap the real CNV) in the middle of the CNV than in the extremes. So, the *margin.pct* parameter defines the percentage of CNV (from each CNV limit) where SNVs will be ignored. By default, only 10% of SNVs from each CNV extreme will be ignored. This *margin.pct* parameter can be modified to better adapt it to your CNV caller. For example, we observed that DECoN produced very accurate CNV calls in our genes panel dataset, so *margin.pct* value was updated to 0 in this context.
- A single reliable SNV can be enough to properly identify false-positive CNV calls, so there is no hard low limit on the number of SNVs required by CNVfilteR. Anyway, CNVs with a bigger number of overlapping SNVs are more likely to be correctly identified.

For other use recommendations and how-to-use guide, visit CNVfilteR vignette at <https://bioconductor.org/packages/release/bioc/vignettes/CNVfilteR/inst/doc/CNVfilteR.html>.
